# Wing Shape Fluctuating Asymmetry in Flies: Insights into Environmental and Public Health Risk

**DOI:** 10.3390/ani15213124

**Published:** 2025-10-28

**Authors:** Hugo A. Benítez, Rocío Oróstica-Pinochet, Manuel J. Suazo, Laura M. Pérez, Jordan Hernández-Martelo, Cristian Valdes, María Teresa Muñoz-Quezada, Margarita Correa

**Affiliations:** 1 Laboratorio de Ecología y Morfometría Evolutiva, Instituto One Health, Facultad de Ciencias de la Vida, Universidad Andrés Bello, República 440, Santiago 8370134, Chile; 2 Research Ring in Pest Insects and Climate Change (PIC2), Santiago 8380453, Chile; 3 Centro de Investigación de Estudios Avanzados del Maule, Universidad Católica del Maule, Talca 3460000, Chile; 4 Cape Horn International Center (CHIC), Centro Universitario Cabo de Hornos, Puerto Williams 6350000, Chile; 5 Vicerrectoría de Investigación y Postgrado, Universidad de La Serena, La Serena 1700000, Chile; 6 Departamento de Ingeniería Industrial y de Sistemas, Universidad de Tarapacá, Arica 1010069, Chile; 7 Programa de Doctorado en Salud Ecosistémica, Centro de Investigación de Estudios Avanzados del Maule, Universidad Católica del Maule, Talca 3466706, Chile; 8 Programa de Epidemiología, Escuela de Salud Pública, Facultad de Medicina, Universidad de Chile, Santiago 8320000, Chile; mtmunoz@uchile.cl

**Keywords:** fluctuating asymmetry, geometric morphometrics, flies, public health, shape variation, developmental stability

## Abstract

**Simple Summary:**

Flies are frequently used as indicators of environmental health because their development is susceptible to stress. In this study, we analyzed wing shape variation to identify subtle asymmetries that occur when organisms are exposed to chemical stressors in agricultural landscapes. Our results indicated that individuals from areas with greater exposure displayed higher levels of asymmetry, which reflects developmental disturbances. This finding demonstrates that wing shape asymmetry can serve as a simple, cost-effective, and reliable method for monitoring the hidden impacts of agricultural chemicals on living organisms and ecosystem health.

**Abstract:**

The widespread but often poorly regulated use of pesticides has triggered urgent debates on their hidden effects beyond resistance in target pests. This study investigates the morphological effects of pesticide exposure, specifically the organophosphate chlorpyrifos, using geometric morphometrics to assess fluctuating asymmetry (FA) in wing shapes of houseflies. Developmental stability (DS), the capacity of an organism to maintain an optimal phenotype under stress, serves as a key indicator of environmental and genetic stress. Flies collected from pesticide-exposed areas in rural areas in Chile (Arbolillo) exhibited significantly higher wing asymmetry than those from less exposed zones, reflecting developmental disturbances caused by chlorpyrifos. These findings emphasize the potential of FA as a biomarker for pesticide-related environmental stress. By linking pesticide exposure to measurable phenotypic disruption, this study calls for urgent integration of morphometric and genomic tools to better understand resistance mechanisms, while also promoting sustainable pest management practices. Our findings demonstrate that even a common insect like the housefly can serve as a biological sentinel, warning of broader ecological and public health risks in pesticide-dominated landscapes.

## 1. Introduction

The housefly, (*Musca domestica*, Linnaeus, 1758), is a cosmopolitan pest of domestic, medical, and veterinary importance [1,2]. It is a synanthropic species, meaning it is closely associated with human settlements and activities [3,4]. The control of the housefly has been a topic of importance in public health. Beyond transmitting diseases, they cause significant annoyance and irritation due to their persistent presence, noisiness, and their habit of contaminating surfaces and food sources, which can lead to a decline in quality of life and sanitation in affected areas. They are potential vectors for over 100 pathogens that impact both humans and animals [5], this species are often abundant in populated areas and livestock breeding sectors such as poultry and cattle farms and are considered a nuisance to humans and animals [2]. In consequence, the relevance of measures to control flies has increased significantly in recent years [6]. Control of this pest has largely relied on chemical methods. Among these, chlorpyrifos is the most widely used chemical compound globally, effective against a wide variety of pests, including rodents, fungi, weeds, insects, and other plant pathogens [7]. The persistence of chlorpyrifos is due to its chemical stability and resilience against natural degradation mechanisms, leading to its extended presence in the environment and accumulation in living organisms, residues in soil, water, vegetables, food items, and human fluids, underscoring its widespread distribution and the possibility of prolonged exposure [8].

According to the Chilean Agricultural and Livestock Service chlorpyrifos is the most widely marketed insecticide in Chile. Although its use was recently restricted (Resolution No. 5810, SAG 2022), this organophosphate remains environmentally persistent and has well-documented neurotoxic and systemic effects on human and animal health [9].

Fluctuating asymmetry (FA) has been extensively studied in other dipterans, offering valuable ecological and functional parallels. For instance, McLachlan [10] demonstrated in chironomid midges that subtle asymmetries in wing length can impair flight performance and mating success, since symmetry is crucial for aerial agility in swarm-based reproductive systems. Parasite infections, particularly ectoparasitic mites, can further increase developmental instability and thus FA, influencing both host aerobatic ability and parasite transmission dynamics. Similar relationships between wing asymmetry, sexual selection, and fitness have been reported in *M. domestica* and other dipterans [11,12], reinforcing the interpretation of FA as a sensitive indicator of physiological stress and overall performance. These comparative examples highlight the functional and evolutionary significance of symmetry within flies and strengthen the rationale for using *M. domestica* as a bioindicator of environmental quality.

Chemical pest control is a contested method due to the rapid development of resistance in target species to one or more chemical groups [5,13]. It has been determined that the frequent and unorganized use of pesticides has been a factor enhancing the unwanted effects of their use, such as the development of resistance in both target and non-target species. Furthermore, it has caused significant environmental problems due to high concentrations of toxins in food, contamination, and persistence in the environment, intoxication of animals and humans, bioaccumulation through the food chain, and negative impacts on soil fertility, among other collateral effects [2,4]. Pesticide resistance has increased worldwide and has become one of the most significant and complex problems in pest control [14,15,16,17]. In particular, the housefly has characteristics that favor the development of pesticide resistance, such as its adaptive capacity to different environmental conditions, high fecundity, short life cycle, and cross-resistance [15]. Resistance to the insecticide pyriproxyfen in *M. domestica* was found to have significant fitness costs. Resistant strains exhibited lower fecundity, reduced larval survival rates, and prolonged developmental times compared to non-resistant strains [18]. These trade-offs highlight the resource allocation challenges between resistance development and biological fitness, suggesting potential avenues for resistance management strategies [19]. Another study compared developmental instability in wild populations (Italy, Slovakia, Spain, and Venezuela) and laboratory colonies (reared at the University of Alicante, Spain) of *M. domestica*. It found that the amount and pattern of wing shape FA, compared among samples within each of the two groups (laboratory and wild), were influenced by genetic and environmental stressors. For instance, genetic bottlenecks due to inbreeding in laboratory colonies and environmental fluctuations in wild populations, such as exposure to insecticides and overwintering stress, significantly contributed to increased FA, reflecting disturbances during development [20]. Fluctuating asymmetry infection by parasites can also induce significant FA during development as it increases developmental stress. This asymmetry negatively impacts traits such as flight performance and mating success in other insect like flies, McLachlan [10] shows that asymmetrical placement of ectoparasitic mites further exacerbates these effects by destabilizing flight and reducing reproductive fitness. The exposure of this species to stress factors such as pesticides, changes in climatic conditions, and different development substrates generate genetic selection pressures, which play a significant role in the genetic and phenotypic variation in the housefly. These developmental and environmental alterations can affect developmental stability, an adaptive capacity of an organism, which can be studied and measured through morphometric tools. Developmental stability (DS) is characterized by an organism’s ability to consistently produce a phenotype according to a predetermined adaptive body plan within a specific set of genetic and environmental conditions [21,22,23,24]. Consequently, it refers to an individual capacity to tolerate developmental disturbances throughout its growth. Tools have been used to detect stress-induced instability by examining the symmetry of morphological traits in individuals (e.g., traditional morphometrics, geometric morphometrics, fluctuating asymmetry) [25,26,27]. The morphological tool most used to detect developmental instability is the Fluctuating asymmetry (FA) [28,29,30,31,32,33], FA is a measure of the small random deviations that occur between the left and right sides of bilaterally symmetrical traits [34]. The adaptation of organisms to the environmental stress such as unfavourable temperatures, chemicals, population densities, and other stressors can increase fluctuating asymmetries [33]. Studying the relationship between phenotype and their external conditions, researchers can test hypotheses regarding the impact of stressors [30,35,36].

Fluctuating asymmetry has also been widely applied as a biomonitoring tool across other insect groups beyond *M. domestica*. For example, several studies on coleopterans have shown that beetle wing asymmetry increases under pesticide exposure and habitat fragmentation, making them useful sentinels of agroecosystem stress [37,38,39,40]. In butterflies, FA has been used to evaluate the effects of heavy metal contamination, urbanization, and climate variability on developmental stability, revealing consistent associations between environmental quality and asymmetry in wing morphology [41]. Similarly, bees have been studied for asymmetry in wing venation, where higher FA has been linked to pesticide exposure, nutritional stress, and pathogen loads, with direct implications for pollination services and ecosystem functioning [42,43,44]. Taken together, these studies highlight that FA provides a broadly applicable framework for assessing hidden environmental disturbances in insects with different life histories and ecological roles, reinforcing its potential as a cross-taxon biomarker of stress.

The Maule Region of central Chile provides a particularly relevant scenario to study developmental instability in relation to pesticide exposure. This territory combines intensive viticulture and agricultural practices, large-scale forestry plantations of *Pinus radiata* and *Eucalyptus* spp., and one of the most extensive pig farming operations in the country. The coexistence of these activities generates overlapping pressures on ecosystems and rural communities, where agrochemical use, habitat transformation, and waste management converge. Previous research in the region has documented the presence of chlorpyrifos metabolites in the urine of schoolchildren living near agricultural fields, highlighting the risks of chronic exposure for human populations [45,46]. In this context, the housefly (*M. domestica*), a synanthropic species closely associated with both livestock facilities and human settlements, becomes a suitable sentinel organism. Assessing wing fluctuating asymmetry in flies from the Maule Region thus represents an opportunity to reveal the hidden impacts of agrochemical practices on both environmental and public health.

It has long been recognized that varying environmental conditions are linked to different levels of stress, which can influence an individual’s capacity to withstand pressure and to modulate the final phenotype. Considering the use of pesticides as a factor altering developmental stability, this study aims to evaluate the adaptive response (developmental instability) of the housefly in different areas of Arbolillo, in the San Javier city, Maule Region, to exposure to organophosphate agricultural pesticides, specifically the use of chlorpyrifos using fluctuating asymmetry tools of the wing shape. Based on the developmental instability framework, we hypothesize that fluctuating asymmetry (FA) will increase in *M. domestica* populations closer to pesticide application areas, reflecting higher exposure to environmental stressors.

## 2. Materials and Methods

### 2.1. Data Acquisition

**Figure 1:**
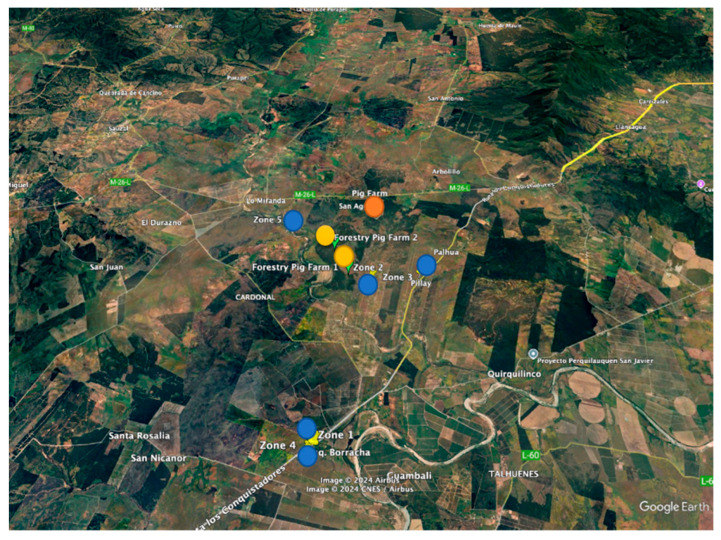
Spatial Distribution of Housefly Collection in the Rural Community of El Arbolillo de San Javier and Nearby Areas in Santo Toribio de Cauquenes, Maule Region, Chile.

229 houseflies (*M. domestica*) were collected from homes in the rural community of El Arbolillo de San Javier and nearby areas belonging to the rural sector of Santo Toribio de Cauquenes in the Maule region, Chile (Figure 1). This area is known for its relevance in beekeeping, viticulture, forestry, and livestock, but it also faces severe socio-environmental conflicts due to the operation of the pig industry.

The vegetation in the study area of San Javier, Maule Region, is characterized by a mix of native and human-altered vegetation types. The natural vegetation includes sclerophyllous shrublands and secondary sclerophyllous forests dominated by native tree species such as hualo (*Nothofagus glauca*). Additionally, the area features Acacia caven steppes and matorral (shrubland) vegetation. These ecosystems have been heavily impacted by human activities, including agriculture, mainly vineyards, firewood production, and intensive silvopastoral systems. Forestry activities are prevalent, with significant portions of the land dedicated to commercial plantations of exotic tree species, primarily Pinus radiata (Monterey pine) and Eucalyptus spp., which are managed intensively for wood and pulp production.

These plantations contribute to the landscape’s composition but also pose environmental challenges, such as reducing biodiversity and altering hydrological cycles. The heavily modified vegetation reflects the dual pressures of agricultural expansion and forestry operations, coupled with socio-environmental conflicts from intensive pig farming and agrochemical usage [45]. The sampling design involved capturing houseflies (*M. domestica*) using adhesive traps deployed in five distinct geographic zones, as identified in Figure 1. These zones were strategically chosen based on their varying distances from the pig farm and two pig farm forestry. The traps were installed in 5 homes across these zones and remained in place for one week to ensure adequate collection of samples. The pollution generated by this facility has significantly impacted air and water quality, affecting the health of the local population and the natural and economic environment [45].

The houseflies were captured inside homes using glue traps installed in five areas (5 traps), distributed between three and ten kilometres away from the animal production plant (pig farm) and two forestry sites owned by the pig farm (see Table 1). This spatial distribution allowed the evaluation of the effects of agricultural pesticides, particularly chlorpyrifos, on the fluctuating asymmetry in the wing morphology of *M. domestica*.

Chlorpyrifos is one of the most widely used insecticides in the Maule Region, primarily for agricultural applications such as vineyards. On the pig farm, insecticides are applied specifically to control fly populations associated with manure management.

Previous studies in the region have detected chlorpyrifos metabolites in the urine of children living and studying near agricultural fields, indicating exposure to this pesticide in communities close to agricultural activities [47]. This study focuses on assessing the presence of chlorpyrifos in flies as an indicator of environmental exposure, bridging agricultural and livestock sources of pesticide use.

### 2.2. Geometric Morphometrics Analyses

**Figure 2:**
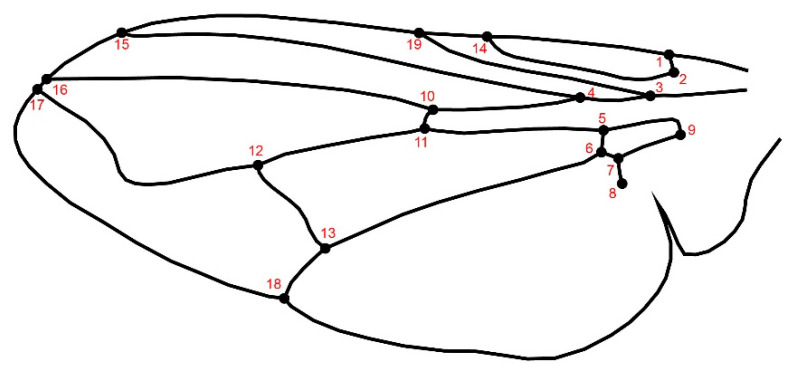
Graphical representation of the wing of *Musca domestica* with 19 landmarks.

Left and Right wings were dissected and mounted using microscope slides, and images were taken using a Stereomicroscope Stemi 305 and Axiocam 208 color (Carl Zeiss Microscopy GmbH, Suzhou, China). 229 samples were used for the morphometrics analyses, 19 landmarks (Figure 2) were digitized on the right and left wings using the software TPS Dig2 v2.31.

Measurement error was considered to avoid the digitizing error on the data and as a vital analysis when fluctuating asymmetry is taken into account [48,49] and a Procrustes ANOVA was calculated where the MS from the error should be smaller than the ones from individual x side (FA).

X and Y landmark coordinates were acquired, and shape information was extracted through Procrustes superimposition analysis [50]. This method aligns the landmark configurations of all analysed individuals, adjusting them to a unit centroid size and eliminating the effects of rotation and translation. Principal Component Analysis (PCA) was then conducted using a covariance matrix of the individual shapes to simulate the shape space and also computed using the average shape in order to identify the differences in shape between localities influenced by the insecticide [51]. Canonical variate analysis (CVA) was used to enhance shape variation and highlight the differences between *M. domestica* localities. Morphological distances, including Mahalanobis and Procrustes distances, were calculated and reported with their respective *p*-values following a permutation test (10,000 rounds). To calculate the Fluctuating asymmetry, a Procrustes ANOVA was performed using the original and repeated data (which are the same data digitized twice) where the interaction individual x side represent the fluctuating asymmetry of the data, and the MS the intensity of the FA, then a multivariate regression was performed using the shape asymmetry data (dependant variable) vs. the Mahalanobis FA score (independent variable) which represent the morphometrics distances from the asymmetric component of the shape.

## 3. Results

**Figure 3:**
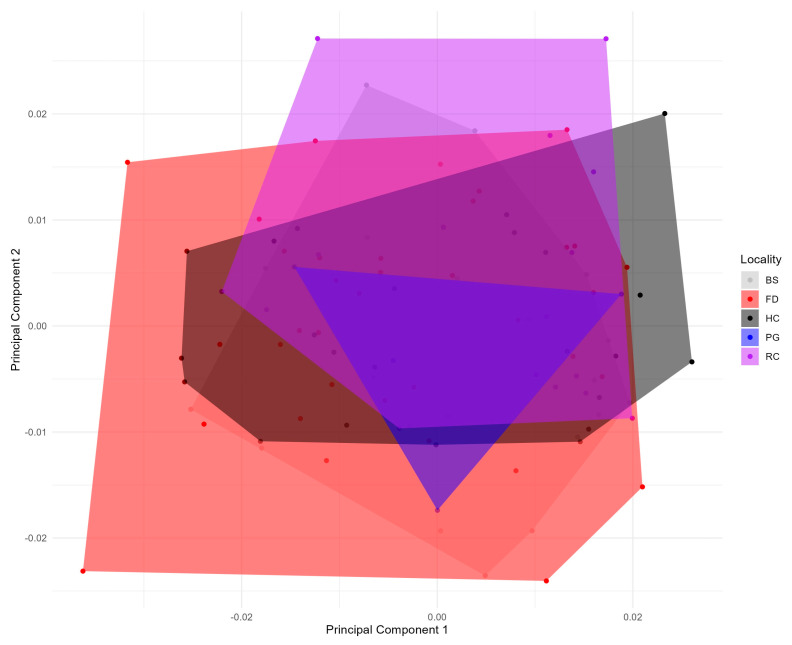
Principal component analysis, of the wing shape of *Musca domestica*, between the five studied zones of the rural area of Arbolillo (colours represent different distances represented at Table 1).

The measurement error of the geometric morphometrics data was found to be neglectable where the MS of the error from the Procrustes ANOVA was smaller than the Individual × side (0.0000010192 < 0.0000051532). The morphospace of the wing in *M. domestica* found that the first three PCs accumulated 56% of the shape variance (PC1: 29.6%; PC2: 15.5%; and PC3: 11.03%). A noticeable superposition of individuals was found for the data analysed (Figure 3) that meant that the distance between multiple houses where the data was taken was not a clear morphological inductor of change.

**Figure 4:**
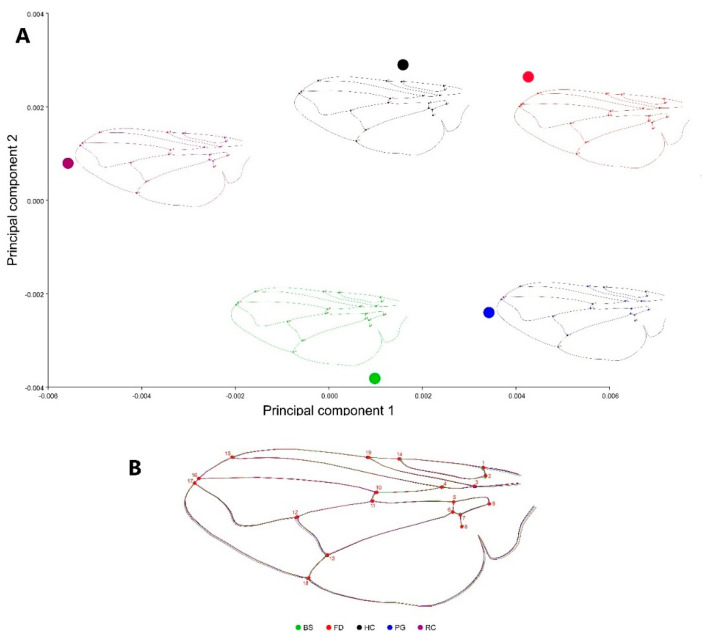
(**A**) Principal component analysis (PCA) of the average wing shape of *Musca domestica* across localities in the Arbolillo region. Each color represents a different locality where traps were placed. (**B**) Wing outlines depict the superimposed average wing shapes for each locality, corresponding to their respective colors.

Nevertheless, a Procrustes ANOVA using the locality as a classifier found that the wing size and shape was significatively different between house distances Centroid Size (F: 3.51, *p*-value: 0.005) and wing shape (Pillay T: 1.77, *p*-value: 0.0004). The average shape PCA demonstrated the positioning of each averaged wing shape within the morphospace of possible shapes. It revealed that the wings differed slightly but significantly among the localities, enabling the identification of distinct morphotypes across the studied transect. The analysis indicated that the cubital vein exhibited greater variability, particularly in the vector movement of landmarks #12 and #13, as well as minor but noticeable variation at landmark #18, located at the end of the anal vein (Figure 4).

**Figure 5:**
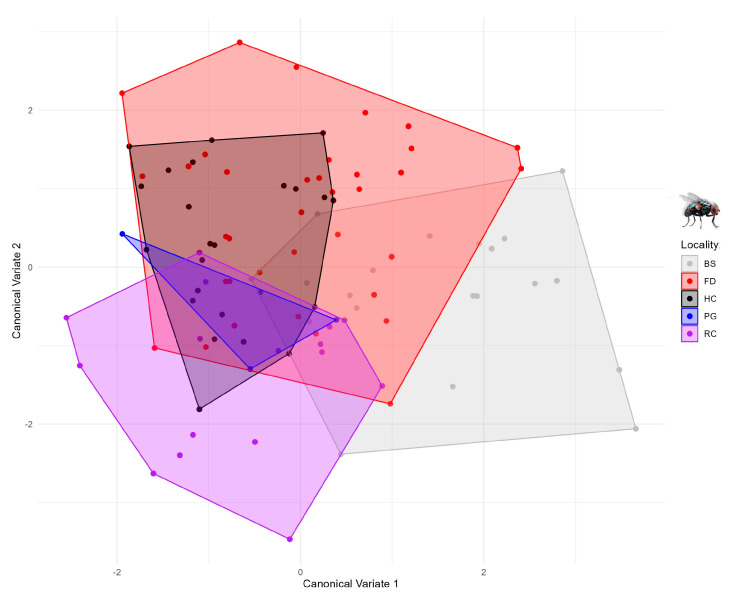
Canonical variate analysis of the wing shape of *Musca domestica* between the five studied zones from the rural area of Arbolillo (colors represent different distances represented at Table 1).

The canonical variate analysis (CVA) enhanced the shape differences among localities and revealed distinct clustering patterns (Figure 5). Individuals from FD exhibited considerable shape variability, overlapping with all other groups, which indicates a high degree of variance within the population. In contrast, flies from BS and RC exhibited more divergent wing shapes, forming clusters that did not overlap with those of the remaining groups, suggesting localized morphological differentiation. Conversely, PG and HC populations displayed strongly overlapping morphospaces, reflecting more similar wing shape configurations across these sites. Overall, the CVA results highlight both shared variation and unique morphotypes among localities, suggesting that exposure intensity and local environmental factors jointly shape patterns of wing morphology.

### Fluctuating Asymmetry

Significant FA levels were found for all the studied localities, both collectively and when analysed independently, where the individual × side factor of the Procrustes ANOVA was highly significant. MS values, which indicate the intensity of FA. When evaluated independently, high levels of FA were found in the PG and FD individuals, while smaller values were found in HC.

**Figure 6:**
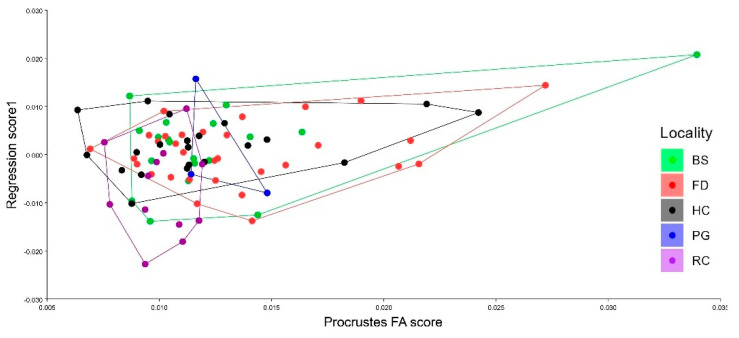
Multivariate regression of asymmetry wing shape of *Musca domestica*, dependent variable are the wing shape and independent variable the Procrustes Scores from the asymmetry which reflect the intensity of the levels of fluctuating asymmetry between the different studied zones.

The multivariate regression of the shape vs. Procrustes FA scores (Figure 6), showed the values of asymmetry were more likely to be from the organism of FD and HD which have more individuals with higher levels of wing shape Fluctuating asymmetry, on the other hand a clear shape variation was found for RC and PG populations, where for the latter a not clear intensity of FA was found, which was not same as found in the Procrustes ANOVA. Finally, a unique organism after (revised any outlier proportion in their asymmetry) was found to have higher levels of FA Scores from BS.

## 4. Discussion

The following research found that geometric morphometrics is a powerful tool to detect levels of fluctuating asymmetry (FA) in *M. domestica* as a measure of developmental instability (DI) across pesticide-exposed environments. Particularly, the FA levels were higher, as expected, in locations closer to the centre. Additionally, shape variance was also found to be greater in individuals closer to the centre.

The absence of a direct correlation between chlorpyrifos concentration and FA intensity likely reflects the multifactorial nature of stress in agricultural landscapes. Beyond pesticide exposure, environmental variables such as ammonia emissions, habitat fragmentation, temperature variation, and nutrient enrichment may act synergistically, affecting developmental stability. Moreover, our study was conducted at a local scale with a moderate sample size, which may have limited statistical resolution for detecting linear associations with chemical concentrations. These results highlight the utility of FA as a sensitive biomarker for environmental stress [30,52,53,54,55]. While the findings reveal significant morphological changes near stressor sites, the lack of a direct correlation between chlorpyrifos concentrations and FA intensity suggests a more complex interaction. Polak [56], mentions that FA’s complex interactions could be related to resistance, insecticide exposure, and fluctuating asymmetry. They analysed ivermectin and observed a significant increase in larva-to-adult mortality in *M. domestica*. Nevertheless, FA in multiple traits of adult flies did not differ between treated and control groups. The lack of observed FA differences was attributed to developmental selection, wherein less developmentally stable individuals failed to survive the insecticide treatment [33]. Morphological differences identified through average shape graph revealed distinct shape profiles in some populations, such as BS, RC, and FD, without clear proximity-based patterns. This indicates that maternal effects or localized environmental variations may play a significant role in shaping these outcomes. Higher levels of FA may impair flight stability, dispersal capacity, and mating success in dipterans, which are essential traits for population resilience and for the maintenance of ecological interactions such as pollination and decomposition [33]. In agricultural landscapes, such developmental disturbances could therefore influence not only the fitness of fly populations but also the ecological services they indirectly support [57]. From a toxicological standpoint, these asymmetric alterations reflect sublethal physiological stress responses, possibly linked to neurotoxic or metabolic disruptions caused by organophosphates. Consequently, the magnitude of FA detected here provides a measurable signal of compromised organismal performance, offering an early-warning indicator of environmental quality under chemical stress This analysis provides an overall picture of the potential consequences and impacts on the fitness of flies inhabiting areas closer to stressors area. Studies of asymmetry are based on the premise that both sides of an organism are independent replicas of the same developmental process. Since both sides share the same genotype and are exposed to a homogeneous environment (i.e., identical on both sides), they are under the influence of the same external factors [22,23,32]. Fluctuating asymmetry (FA) is considered a measure of developmental instability (DI) based on the idea of detecting and quantifying how these perturbations act locally, affecting a particular part of the body [23,31]. This sensitivity to random perturbations, also known as developmental noise, reflects the tendency of a developmental system to generate morphological changes in response to such perturbations [35,38]. Chlorpyrifos application was measured in the insects, and the tendency was not directly related to the asymmetry levels detected; nevertheless, the observed shape variation showed a clear intensity of variation on the morphospace for population, which inhabited zones closer to the animal centre, and less and more superposed variation was detected on the other zones. The average shape PCA (Figure 4), results identify particular groups of wing shape variation, BS, RC, and FD, without a clear tendency to correlate with the proximity to pesticide origin. Vilaseca et al. [58] demonstrated that long-term exposure to pyrethroid insecticides, such as deltamethrin and alpha-cypermethrin, could lead to persistent environmental residues and potential resistance in insect populations where individuals of *T. infestans* from Bolivian populations in Yamparáez/Sotomayor, after 17 years of biannual chemical treatments, showed high AF patterns, but little morphometric variation, inducing enhanced levels of resistance in individuals, unlike insects collected with only 1–2 years of insecticide application where residual effects of these treatments were evaluated with greater variation in the wings of Huacaya/Imbochi triatomines. On the other hand, Floate and Fox [59] aimed to examine the relationship between stress, fitness, and fluctuating asymmetry (FA) in laboratory populations of house flies (*M. domestica*) where analysis revealed no significant differences in FA levels across five different wing traits among the populations studied their findings indicate that FA may not be a reliable method for assessing changes in environmental quality. Clarke and Ridsdill-Smith [60], analysed the exposure of AvermectinB1 in the treatment on Cattle, on normal developmental processes in the bush fly, *Musca vetustissima* finding higher levels of FA on flies which come from the pesticide application on two morphometric traits (wing vein lengths), flies breeding in dung from untreated cattle or cattle treated with Levamisole-HCl were found to not being asymmetric. This suggests that the effects of environmental stressors like insecticides on FA can be masked by selective mortality, highlighting the complexity of using FA as a biomonitoring too. Our specific results indicated that the use of geometric morphometrics to identify wing asymmetry was a highly sensitive method, revealing clear levels of asymmetry in *M. domestica* individuals that appear to be more exposed to chlorpyrifos. The absence of a direct correlation between chlorpyrifos concentration and FA intensity may be the result of several interacting mechanisms. First, flies are exposed to a combination of environmental stressors in rural settings, including ammonia emissions from animal waste, nutrient runoff, and temperature fluctuations, all of which can act synergistically with pesticides to alter developmental stability. Second, cumulative exposure to sublethal doses across generations could produce non-linear or delayed morphological responses, masking direct associations in cross-sectional data. Finally, selective mortality may reduce the apparent FA signal, as individuals with extreme developmental instability might not survive to adulthood. Such interactions between chemical and non-chemical stressors have been documented in other dipteran studies, highlighting that FA in field populations often reflects the combined outcome of multiple selective pressures rather than a single contaminant effect. However, the multivariate regression showing higher FA scores in individuals from HC, which is the second farthest zone (8.7 km from the center), suggests that most of the area could be affected. These results suggest that FA not only serves as a tool for detecting immediate stress effects but also provides insights into the potential for long-term environmental impacts. These findings highlight the need for integrating morphometric and genomic approaches to unravel resistance dynamics and stress response mechanisms in populations exposed to agricultural chemicals. The observed increase in FA in flies inhabiting areas closer to agricultural and livestock facilities suggests that environmental stress is not confined to insects but may also reflect broader ecological disruption. Within a One Health perspective, such developmental instability can act as an early indicator of environmental degradation that may affect animal and human populations through shared exposure pathways. Similar studies in the Maule Region have documented organophosphate metabolites in children living near agricultural fields, supporting the use of sentinel species like *M. domestica* to bridge environmental and public health monitoring. It is important to mention that this study was conducted over a relatively small spatial scale, which may limit the generalizability of the findings. Future research should include larger spatial scales, additional taxa, and expanded environmental variables to fully understand the interplay between pesticide exposure, resistance development, and developmental stability. Furthermore, incorporating genomic tools to investigate selection pressures and resistance-related genes could provide a more comprehensive understanding of how populations adapt to chronic stress.

## 5. Conclusions

This study demonstrates that fluctuating asymmetry (FA) in the wings of houseflies, measured using geometric morphometrics, is a sensitive indicator of developmental instability following exposure to agricultural chemicals. Higher asymmetry in populations near stressor sites highlights FA as a reliable biomarker of environmental stress, even when direct correlations with chlorpyrifos concentrations are not straightforward.

The results underscore the importance of combining morphometric and genomic approaches to understand resistance dynamics and adaptive responses better. Beyond its scientific contribution, this work emphasizes the importance of developing sustainable pest management strategies that balance ecosystem health and public well-being. In practical terms, incorporating FA monitoring into environmental assessment programs could help detect early sublethal effects of chemical stress before visible ecological damage occurs. This approach offers policymakers a cost-effective and biologically meaningful tool for evaluating the sustainability of agricultural practices and mitigating public health risks.

## Figures and Tables

**Table 1:** Sampling zones in the locality of El Arbolillo, Maule Region, where *Musca domestica* were collected, distances are in meters.

Zone	Code	Distance to Centre (m)	Distance to Forestry (m)	Distance to Forestry 2 (m)
Zone 1	HC	8798	5897	6806
Zone 2	RC	5702	7677	7651
Zone 3	PG	3235	2312	3001
Zone 4	BS	9113	6220	7114
Zone 5	FD	3160	2821	1873

## Data Availability

Data available on demand to the corresponding authors.
